# Household

**Published:** 1889-09

**Authors:** 


### HOUSEHOLD.

RASPBERRY Jam.—To each pound of fruit allow 1 lb. of ground loaf-sugar, pick the berries carefully, put them in a preserving-pan, or an enamelled one, along with the sugar; put the pan on a clear brisk fire, and with a wooden spoon stir carefully from the bottom, as it is very apt to burn. When it comes to the boil, let it boil quickly eight minutes, stirring all the time; draw the pan to the side of the fire, skim it carefully, and pot it. When cold cover up nicely. As raspberries very soon turn mouldy after they are gathered, it is most important that fresh fruit should be used, and the jam stored in a dry, airy place.

RASPBERRY JAM AND JELLY.—Have equal weights of raspberries and red currants; pick the currants from the stalks, and allow 1 lb. of sugar to each pound of fruit; put all in the pan together, and stir with a wooden spoon until it boils. Skim it carefully when boiling; boil it twenty minutes. When done, run a few pots of the jelly through a piece of muslin, and return the berries that are in the muslin into the pan, and pot it up. When cold cover up carefully.

RASPBERRY WINE.—(1) Bruise some ripe raspberries picked free from husks, add an equal quantity of water, and let them stand for two days. Squeeze them through a bag, and to every gallon of juice add 3 lb. of sugar. Put the juice into a cask, and when it has done fermenting, add half a pint of spirits or a pint of white wine to every gallon. Bung it up close, keep in a cool place, and bottle in four months. (2) To every quart of berries, gathered in fine weather, and freed from the husks, put the same measure of good cider, let it steep two days; then press out the juice, and to each quart put ¾ lb. of sugar. Rinse a cask out with brandy, put the juice into it, and to every gallon put half a pint of brandy. In three months, bottle and cork well. Fit for use at Christmas.

CHANGE IN EGGS.—It is a common fact that eggs, though so well enclosed, begin to decrease in specific gravity as well as freshness, as soon as they meet the air. This is shown by the popular test of age in lye or brine. A fresh-laid egg will descend to the bottom of a solution that will float it higher every day thereafter, until it will soon appear above the surface. A coating impervious to the air prevents these changes.

WHAT TO DRINK.—Instead of drinking so much green and black tea, that tends to make women wakeful, nervous and irritable, try beef tea. Take lean meat, cut it up small, and boil several hours; skim off the grease, and serve hot and well-seasoned in cups. One with this substitute will hardly miss her foreign tea, often sadly adulterated and rarely quite sure, and she will be astonished at the gain in nerve, freedom from irritability, and at the good sound sleep she will have every night.

---

We may earnestly pray that the proehecy of Victor Hugo shall be fulfilled, and speedily, "In the twentieth century war will be dead, the scaffold will be dead, but man will live."

